# Efficacy of Serratus Posterior Superior Intercostal Plane Block (SPSIPB) in Cardiac Implantable Electronic Device Implantation (CIED): A Prospective, Double-Blind, Randomized Controlled Trial

**DOI:** 10.3390/jcm15145629

**Published:** 2026-07-17

**Authors:** Gozde Altun, Ayla Esin, Yasemin Ozsahin, Sukru Arslan, Mehmet E. Bilgin, Baris Sandal, Kerem Erkalp, Ziya Salihoglu

**Affiliations:** 1 Department of Anesthesiology and Reanimation, Institute of Cardiology, Istanbul University-Cerrahpaşa, 34096 Istanbul, Türkiye; 2 Department of Cardiology, Institute of Cardiology, Istanbul University-Cerrahpaşa, 34096 Istanbul, Türkiye; 3 Department of Biostatistics, Cerrahpaşa Faculty of Medicine, Istanbul University-Cerrahpaşa, 34320 Istanbul, Türkiye; 4 Department of Anesthesiology and Reanimation, Cerrahpaşa Faculty of Medicine, Istanbul University-Cerrahpaşa, 34098 Istanbul, Türkiye

**Keywords:** cardiac electrophysiology, cardiac implantable electronic devices, postoperative analgesia, serratus posterior superior intercostal plane block, satisfaction, sleep quality

## Abstract

**Background:** This study evaluated the analgesic efficacy of the serratus posterior superior intercostal plane block (SPSIPB) in patients undergoing CIED implantation. **Methods**: In this study, 60 patients undergoing primary CIED implantation were randomized to receive either ultrasound-guided SPSIPB (*n* = 30) or a sham-control group procedure (*n* = 30). The primary outcome was perioperative pain intensity, assessed using the Visual Analog Scale (VAS) intraoperatively and at 3, 6, 12, and 24 h postoperatively. Secondary outcomes included postoperative sleep quality measured by the Sleep Quality Numeric Rating Scale (SQ-NRS), patient and physician satisfaction scores. **Results**: SPSIPB significantly reduced pain scores at all time points compared with the control group (all *p* < 0.001). Clinically significant intraoperative pain (VAS ≥ 4) occurred in 83.3% of controls but in none of the SPSIPB patients (*p* < 0.001). Postoperatively, clinically significant pain dropped from 93.3% in controls to just 3.3% with SPSIPB (*p* < 0.001), with the block group maintaining significantly lower pain scores at 3, 6, 12, and 24 h (mean reductions vs. control of 4.70, 4.13, 3.97, and 1.90 points, respectively; *p* < 0.001). SQ-NRS scores were lower in the SPSIPB group than in the control group (median 2 vs. 7, *p* < 0.001). Poor sleep quality (SQ-NRS ≥ 6) was observed in 80.0% of controls and in none of the SPSIPB patients. Patient and physician satisfaction were significantly higher in the SPSIPB group (median Likert score: 5 vs. 3, *p* < 0.001). **Conclusions**: Preoperative SPSIPB was associated with substantially lower perioperative pain scores and reduced rescue analgesic requirements during the first 24 h after CIED implantation. Improvements in postoperative sleep quality and patient–physician satisfaction were also observed; however, these secondary outcomes should be interpreted as exploratory. Larger multicenter trials are warranted to confirm the magnitude and generalizability of these findings.

## 1. Introduction

Cardiac implantable electronic devices (CIEDs) serve a critical role in managing arrhythmias, enhancing patient clinical status, and preventing sudden cardiac death [1]. The spectrum of CIEDs encompasses pacemakers (PMs), implantable cardioverter-defibrillators (ICDs), and cardiac resynchronization therapy (CRT) systems. Structurally, these devices are placed in the left or right infraclavicular region, just anterior to the anterior axillary line, which corresponds approximately to the T2–T3 dermatomes [2]. The number of CIED implantations performed worldwide has increased steadily over recent decades. Along with this expanding use, peri-procedural and long-term device-related complications have also become more frequent and clinically relevant. These complications include pocket hematoma, pneumothorax, lead-related problems, and device infection [3]. Therefore, optimizing perioperative management, including effective analgesia and procedural safety, is increasingly important in patients undergoing CIED implantation.

Patients scheduled for CIED implantation typically present with a high burden of comorbidities. Consequently, perioperative anesthesia and analgesia management demand meticulous attention and structured planning [4]. In recent years, numerous centers have successfully performed CIED implantations by combining local anesthesia with conscious sedation [5,6].

Ensuring effective perioperative anesthesia and analgesia is paramount for patients scheduled for CIED implantation. Inadequate intraoperative local anesthesia frequently leads to pain perception during needle puncture and pocket creation. The involuntary patient movements triggered by this pain can undermine procedural precision, potentially forcing the clinician to perform multiple puncture attempts. Furthermore, such intraoperative instability increases the risk of procedural complications, including bleeding and pneumothorax [7,8].

Given that the patient population undergoing CIED implantation is predominantly elderly with a high prevalence of cardiac risk factors and comorbidities, postoperative pain can substantially exacerbate myocardial stress. This physiological strain potentially triggers adverse hemodynamic fluctuations, making perioperative analgesia a critical priority in this group [9]. Furthermore, managing postoperative pain in CIED patients presents a unique challenge for clinicians, driven by highly variable patient expectations, previous negative healthcare experiences, the severity of underlying diseases, and individual variations in pain sensitivity [10].

In clinical practice, conventional intravenous analgesics administered during the postoperative period may not achieve sufficient efficacy or suitability for every patient. A distinct correlation exists between heightened postoperative pain sensitivity and sleep disturbances. Crucially, postoperative sleep quality exerts a direct influence on both pain perception and overall recovery, while also serving as a key determinant of patient satisfaction [11,12,13]. In contrast, newly emerged fascial plane blocks offer versatile applications and can enhance both patient and physician satisfaction by providing more sustained and adequate analgesia.

The serratus posterior superior intercostal plane block (SPSIPB) is a newly described regional technique utilized to achieve hemi-thoracic analgesia [14]. Accumulating evidence demonstrates that SPSIPB provides safe and effective analgesia across various surgical spectrums—including video-assisted thoracoscopic surgery, breast cancer surgery, clavicle surgery, and minimally invasive cardiac procedures—as well as in the management of chronic pain [15,16,17,18,19].

This study aimed to evaluate the analgesic efficacy of SPSIPB in patients undergoing CIED implantation by assessing intraoperative and postoperative 24 h pain scores using the Visual Analog Scale (VAS). Secondary objectives included evaluating postoperative sleep quality via the Sleep Quality Numeric Rating Scale (SQ-NRS) and measuring both patient and clinician procedural satisfaction using a 5-point Likert scale.

## 2. Materials and Methods

### 2.1. Study Population

This prospective, double-blind, randomized controlled trial was conducted at the Department of Anesthesiology and Reanimation, Istanbul University-Cerrahpaşa, Institute of Cardiology, between September 2025 and April 2026. The study enrolled patients evaluated in the institute’s cardiac electrophysiology laboratory. Institutional ethics approval was granted by the Istanbul University-Cerrahpaşa, Medical Research Ethics Committee (Approval number: E-24687260-804.01-1395253), and the trial protocol was registered at clinicaltrials.gov (NCT07165041, 2 September 2025). The study design and reporting adhered to the Consolidated Standards of Reporting Trials (CONSORT) guidelines [20]. The completed STROBE checklist is provided as a Supplementary Materials File. Throughout the study period, all investigators strictly complied with the principles outlined in the Declaration of Helsinki. Prior to participation, written informed consent was obtained from all patients.

Patients aged 18 years or older who provided written informed consent, those undergoing primary CIED implantation, and individuals with no documented clinical infection or hemostatic disorders were included in the study.

Conversely, the exclusion criteria comprised patients with advanced decompensated heart failure (New York Heart Association [NYHA] Class IV), known hypersensitivity to the study medications, morbid obesity (body mass index [BMI] > 35 kg/m^2^), or an active infection/lesion at the scheduled block site. Additionally, patients with severe communication barriers, inability to tolerate the required positioning for the block, diagnosed psychiatric disorders, progressive neurological or muscular diseases, an established history of chronic pain, or diagnosed sleep disorders requiring active treatment (e.g., obstructive sleep apnea syndrome, insomnia) were excluded. Finally, individuals undergoing CIED revision, device upgrade, or battery replacement, as well as those who declined to participate, were excluded from the trial.

### 2.2. Randomization and Blinding

Patients were randomly assigned in a 1:1 ratio into two equal groups of 30 individuals each: the SPSIPB group (Block group) and the control group. Random assignment sequences were generated using an online platform (Randomizer.org, Dublin, Ireland) to ensure equitable distribution between the groups.

Patients in the block group received the SPSIPB intervention thirty minutes prior to the scheduled CIED implantation. For those in the control group, a sham procedure mimicking the exact sequence of the block group was performed thirty minutes before the operation, using a minimal volume of saline (1 mL) instead of the local anesthetic agent. The anesthesiologist performing the block was unblinded to the allocation. However, the patients and the operating cardiologists remained strictly blinded to the group assignments throughout the trial. Furthermore, a separate anesthesiologist, who was completely blinded to the study protocol, conducted all postoperative assessments, including the evaluation of pain scores via the VAS, sleep quality via the SQ-NRS, and patient and physician satisfaction.

### 2.3. Interventional Procedure

In accordance with the institute’s standard protocol, intravenous sedation is routinely omitted during CIED implantations; therefore, no sedation was administered to patients in either group. Strict adherence to aseptic techniques was maintained for all patients. The block procedures were performed under ultrasound guidance by a single anesthesiologist highly experienced in regional anesthesia (>10 years). To ensure consistency, patients from both groups were transferred to a dedicated procedure room, where the block was executed 30 min prior to the implantation. Before the intervention, all patients were informed about alternative pain-management strategies available in the event of inadequate analgesia. Additionally, standard resuscitation and emergency equipment were kept fully operational and readily accessible during all procedures.

#### 2.3.1. SPSIPB Procedure (Block Group)

Upon arrival in the procedure room, patients were monitored in accordance with the American Society of Anesthesiologists (ASA) standards, including non-invasive blood pressure, peripheral oxygen saturation, and 5-lead electrocardiogram. The patients were placed in a sitting position. To optimize sonographic visualization, the arm on the procedural side was gently pulled across the torso into adduction and internal rotation, thereby displacing the scapula laterally. After ensuring strict asepsis of the target area, skin infiltration was performed at the needle insertion site using 1% lidocaine.

**Figure 1:**
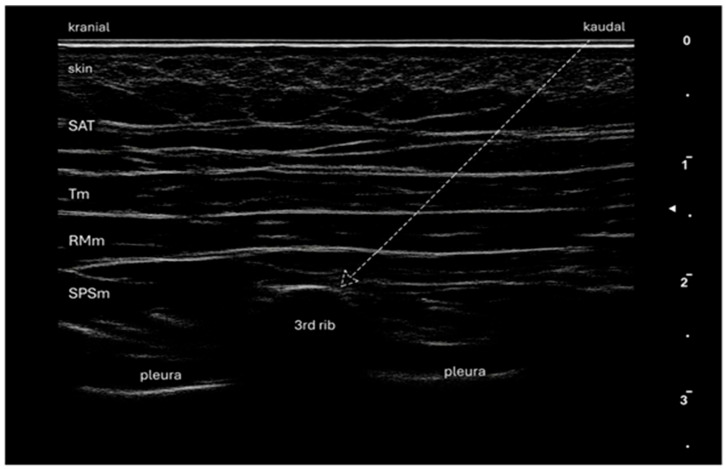
Ultrasound image demonstrating the serratus posterior superior intercostal plane block (SPSIPB). SAT: subcutaneous adipose tissue; Tm: trapezius muscle; RMm: rhomboid major muscle; SPSm: serratus posterior superior muscle. The dashed arrow indicates the path of the needle and its target point under ultrasound guidance.

Ultrasound imaging was conducted using a Philips Epiq 7 system equipped with a linear 4–12 MHz transducer (Philips Ultrasound; Bothell, WA, USA). For the SPSIPB approach, the linear probe was positioned longitudinally over the superomedial aspect of the scapula to identify the first rib. The transducer was then translated caudally while counting the ribs to pinpoint the second and third intercostal spaces. At this level, the trapezius, rhomboid major, and serratus posterior superior (SPS) muscles were clearly visualized (Figure 1). A 22-gauge, 80 mm peripheral block needle (Echoplex+, VYGON, Paris, France) was advanced in a caudo-cranial direction through the skin, subcutaneous tissue, and adipose layers. After contact of the needle with the 3rd rib gently, 1–2 mL of saline was used to confirm the correct plane, and a total of 30 mL of 0.25% bupivacaine was administered to the superficial to the intercostal muscle and the caudo-cranial spread of the local anesthetic was dynamically observed [14].

#### 2.3.2. Sham Procedure (Control Group)

To rigorously maintain the double-blind nature of the trial, patients allocated to the control group underwent an identical preparation and procedural sequence to mimic the experience of the block group. Patient monitoring and positioning were executed in the exact same manner as previously described. Using ultrasound guidance, optimal visualization of the target interfascial anatomy was achieved, followed by skin infiltration with 1% lidocaine at the designated insertion site.

The 22-gauge, 80 mm block needle was then advanced utilizing the identical technique detailed for the block group. Upon successfully navigating the needle tip into the interfascial space between the rhomboid major and SPS muscles, a minimal volume of 1 mL of saline was injected to finalize the mock intervention. Crucially, no local anesthetic agents were administered into the interfascial plane in this patient group.

### 2.4. Assessment of Block Success

Thirty minutes after the intervention, patients from both groups were transferred to the cardiac electrophysiology laboratory. Prior to the administration of any local anesthetic by the operating cardiologist, a cold sensation test was performed using an alcohol-based disinfectant to evaluate sensory blockade. Sensory loss was assessed across the dermatomes potentially covered by the SPSIPB and compared with a non-blocked reference area. The block was defined as successful if the patient reported either diminished or completely absent cold perception in the target dermatomes relative to the control region [21].

### 2.5. Surgical Procedure for CIED Implantation

Thirty minutes following the interventional block, fully monitored patients from both groups were escorted to the cardiac electrophysiology laboratory. All implantations were executed by a single, high-volume cardiologist with extensive experience in device therapy (>100 CIED implantations annually). In accordance with the institutional standard of care, local field infiltration at the puncture site and the designated device pocket area was performed using 20 mL of 2% prilocaine (Priloc 2%, 20 mL, Vemilaç, Istanbul, Turkey). A 5 min interval was observed to allow for adequate onset of local anesthesia.

Subsequently, venous access for lead advancement was secured via axillary or subclavian vein puncture. For the creation of the generator pocket, a 3-to-5 cm incision was made parallel to and approximately 5 to 10 cm inferior to the clavicle. Blunt dissection was carefully carried out to develop either a subfascial or subpectoral pocket. Following lead positioning verification and connection to the generator, the device was secured within the pocket, and the surgical site was closed anatomically according to standard protocols. Postoperatively, all patients were transferred to the coronary care unit (CCU) for a 24 h intensive monitoring period. Upon completion of CCU surveillance, patients were transferred to the inpatient ward for an additional two days of observation prior to hospital discharge.

### 2.6. Data Collection

Baseline demographic characteristics, including age, sex, body mass index (BMI), educational background, and relevant comorbidities, were recorded for all participants. Additionally, procedure-related variables were documented, specifically the venous access site utilized (axillary or subclavian vein), device category (PM, ICD, or CRT), number of leads implanted, orientation of CIED placement (right or left hemisphere), and anatomical depth of the generator pocket (subcutaneous, subfascial, or subpectoral). The technical difficulty of venous puncture was categorized dichotomously, with two or more attempts defined as a difficult puncture.

Intraoperative parameters, such as total fluoroscopy duration and the cumulative dose of local anesthetics administered, were meticulously logged. Postoperatively, the specific class and total consumption of rescue analgesics were tracked. Furthermore, any adverse events and procedural complications encountered throughout the perioperative period were systematically monitored and recorded.

#### 2.6.1. Pain Assessment and Rescue Analgesia Protocol

Patient pain levels were meticulously evaluated using the Visual Analog Scale (VAS) intraoperatively and at the 3rd, 6th, 12th, and 24th postoperative hours. Prior to the procedure, the mechanics of the 10 cm VAS line were explained to each participant in detail. Patients were instructed that a score of 0 represented the complete absence of pain, 5 signified moderate pain, and 10 denoted the most severe pain imaginable. Pain scores were determined by asking patients to mark their perceived pain intensity along this 10 cm continuum [22].

Strict intraoperative and postoperative rescue analgesia algorithms were established for both groups based on these scores. During the surgical implantation, if a patient reported a pain score of VAS ≥ 4, first-line rescue analgesia was initiated by the operating cardiologist via local infiltration of 100 mg (5 mL) of prilocaine into the painful area. If the patient experienced recurrent breakthrough pain with a subsequent score of VAS ≥ 4, a second and final intraoperative rescue dose of 100 mg (5 mL) of prilocaine was administered.

In the postoperative period, a standardized multimodal analgesic escalation protocol was implemented. For any documented pain score of VAS ≥ 4, first-line rescue analgesia consisted of 1 g of intravenous paracetamol (Partemol^®^ 1 g/100 mL; Vemilaç, Istanbul, Turkey) administered as a 20 min infusion. If the pain remained refractory with a VAS ≥ 4 at least two hours following the paracetamol infusion, second-line rescue analgesia was provided via intravenous administration of 50 mg of tramadol HCl (Tradolex^®^ 100 mg/2 mL; Mentha Pharma, Istanbul, Turkey). For ongoing postoperative pain management, scheduled maintenance doses of intravenous 1 g paracetamol were permitted as needed for scores of VAS ≥ 4, provided that a minimum dosing interval of 6 h was strictly maintained.

#### 2.6.2. Assessment of Sleep Quality and Procedural Satisfaction

Postoperative sleep quality was evaluated using the Sleep Quality Numeric Rating Scale (SQ-NRS), a patient-reported outcome measure designed to quantify subjective sleep disturbances. On this 11-point numeric scale, a score of “0” was defined as “excellent sleep,” whereas a score of “10” signified “complete inability to sleep throughout the entire night.” The clinical implications of each numerical value were thoroughly explained to all participants. On the first postoperative morning, patients from both groups were questioned regarding their sleep experience from the preceding night, with an SQ-NRS score of ≥6 classified as poor sleep quality [23].

To evaluate procedural satisfaction, a 5-point Likert scale was utilized for both patients and clinicians. The scale anchor points were carefully described to the participants, where a score of 1 corresponded to “not at all satisfied” and a score of 5 denoted “very satisfied” [24]. On the first postoperative day, satisfaction levels were independently reported by the patients and the cardiologist, and the respective scores were documented for analysis.

### 2.7. Study Outcomes

The primary outcome of this study was the assessment of perioperative pain intensity, quantified using the Visual Analog Scale (VAS) intraoperatively and at predetermined postoperative intervals (the 3rd, 6th, 12th, and 24th hours) in patients undergoing CIED implantation with preoperative SPSIPB.

The secondary outcomes comprised the evaluation of subjective sleep quality during the first postoperative night via the Sleep Quality Numeric Rating Scale (SQ-NRS), alongside the measurement of procedural satisfaction levels for both patients and clinicians using a 5-point Likert scale.

### 2.8. Statistical Methodology and Sample Size

#### 2.8.1. Sample Size Estimation

The required sample size for comparing pain intensity, assessed using VAS scores, between the SPSIPB and control groups was determined using G*Power software version 3.1.9.7 (Heinrich-Heine-Universität Düsseldorf, Düsseldorf, Germany) based on an independent-samples *t*-test configuration for two-group mean comparisons. The primary estimation assumed a two-tailed distribution with an alpha (α) level of 0.05 and a statistical power (1-β) of 80%.

Because no previous study had evaluated SPSIPB specifically in patients undergoing CIED implantation, the sample size calculation was necessarily based on indirect evidence from studies of comparable fascial plane blocks used in thoracic or chest-wall procedures [25,26,27]. Given the exploratory nature of this clinical application and the absence of procedure-specific preliminary data, Cohen’s d = 0.8 was used as a pragmatic planning assumption rather than as a definitive estimate of the expected treatment effect. Based on this assumption, the minimum required sample size was 26 patients per group. To compensate for potential dropouts, data loss, or unexpected exclusions, 60 patients were enrolled in total, with 30 patients allocated to each group.

#### 2.8.2. Statistical Analysis

All statistical computations were executed using IBM SPSS Statistics for Windows, version 31.0 (IBM Corp., Armonk, NY, USA). The distributional normality of continuous data was formally verified via the Shapiro–Wilk test. Accordingly, normally distributed continuous variables are expressed as mean ± standard deviation, whereas heavily skewed or non-normally distributed metrics are summarized as median with interquartile range [Q1–Q3]. Categorical data are presented as absolute frequencies alongside their corresponding percentages.

Inter-group comparisons were tailored to specific variable types and underlying data distributions. For parametric continuous vectors, the independent-samples *t*-test was applied; conversely, the non-parametric Mann–Whitney U test was utilized to evaluate ordinal or non-normally distributed continuous indices. Proportional variations among categorical elements were analyzed using Pearson’s chi-square test, Fisher’s exact test, or the Fisher-Freeman-Halton exact test, based on cell frequency requirements.

To capture the clinical burden of pain, a VAS threshold of ≥4 was employed to define clinically meaningful pain endpoints. Intraoperative clinical pain was derived directly from the operative VAS log, whereas clinically significant postoperative pain was established if a patient registered a VAS score of ≥4 during at least one of the longitudinal assessments (at the 3rd, 6th, 12th, or 24th hour). The relative risk for developing significant postoperative pain was estimated in conjunction with its 95% confidence interval (CI).

Longitudinal fluctuations in VAS trajectories were mapped via a two-way mixed-design repeated-measures analysis of variance (RM-ANOVA), assigning the intervention group as the between-subject factor and the time sequence as the within-subject factor. Structural sphericity was explicitly checked using Mauchly’s test, and in instances where this assumption was violated, the Greenhouse–Geisser adjustment was implemented. Statistical effect sizes for these trends were quantified using partial η^2^ values.

For deeper exploratory insights into pain profiles, secondary summary metrics—namely the postoperative mean VAS, maximum postoperative VAS, and time-weighted postoperative area under the curve (VAS-AUC)—were derived from the multi-point postoperative timeline. Spearman’s rank correlation coefficients were computed to examine potential linkages between these summary pain dimensions and baseline clinical or procedural markers (age, BMI, subcutaneous fat thickness, fluoroscopy exposure time, and venous access difficulty). Furthermore, stratified exploratory cross-comparisons of pain summary measures against venous puncture difficulty were conducted utilizing the Mann–Whitney U test, while the clinical pain prevalence was mapped via Fisher’s exact test.

Finally, group-adjusted linear regression frameworks were modeled to isolate the simultaneous predictive influence of treatment allocation, subcutaneous fat thickness, venous puncture difficulty, and BMI on both mean and peak postoperative VAS outcomes. The resulting regression coefficients (β) are reported alongside their 95% CIs. Within these systems, subcutaneous fat thickness entered the equation as a continuous vector, whereas venous puncture difficulty was introduced as a binary predictor.

For all analytical frameworks, statistical testing was two-tailed, with a *p*-value of <0.05 denoting nominal significance. No formal multiplicity adjustment was applied for secondary or exploratory analyses. Therefore, *p* values for SQ-NRS, satisfaction scores, analgesic consumption, and exploratory association models should be interpreted as nominal, and these findings should be regarded as hypothesis-generating rather than confirmatory.

## 3. Results

### 3.1. Baseline and Procedural Characteristics

**Figure 2:**
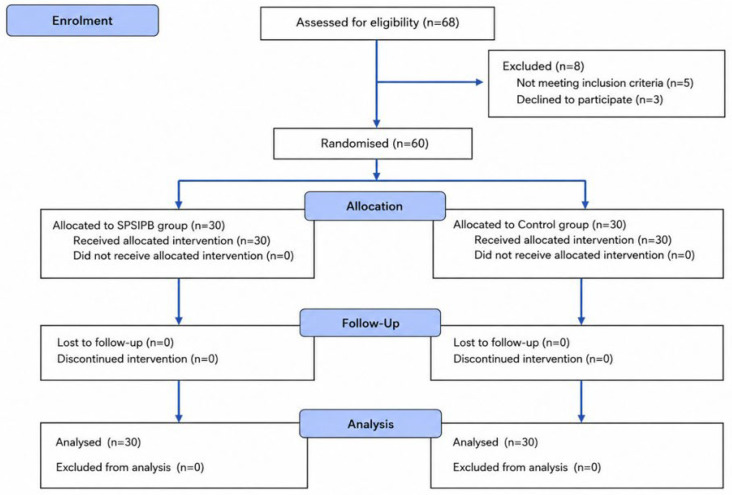
Flow chart of the study.

The final analysis population comprised 60 patients, distributed equally with 30 individuals assigned to the control group and 30 to the block group (Figure 2).

Detailed baseline demographic and clinical features are documented in Table 1. Within the overall study population, the median age was 64.5 [58.3–75.8] years. Stratified by group, the median age was 61.5 [51.3–74.5] years for the control group and 70.5 [60.8–77.3] years for the block group; while the patients receiving the block leaned older, this demographic variance did not achieve statistical significance (*p* = 0.071).

Table 2 outlines the operational and procedural characteristics of the study. Notably, the total volume of supplemental local anesthetic (LA) administered intraoperatively was significantly lower in the block group than in the control group (20.0 [20.0–20.0] mL vs. 25.0 [23.8–30.0] mL; *p* < 0.001). Regarding the safety profile, procedure-related complications were statistically comparable between the two groups (*p* = 1.000), developing in 4 patients (13.3%) in the control group following the mock intervention and in 3 patients (10.0%) in the intervention group after the SPSIPB procedure. Specifically, adverse events within the SPSIPB group included transient vagotonia (*n* = 2) and isolated nausea (*n* = 1) during block execution. Conversely, complications in the control group manifested as nausea during the sham needle insertion (*n* = 2) and vagotonia secondary to acute, implantation-induced pain (*n* = 2).

### 3.2. Pain Profiles, Sleep Quality, Satisfaction, and Analgesic Consumption

Comprehensive clinical outcomes regarding pain intensity, subjective sleep quality, satisfaction metrics, and analgesic requirements are consolidated in Table 3.

Throughout the entire longitudinal monitoring framework—spanning the intraoperative stage through the 3rd, 6th, 12th, and 24th postoperative hours—VAS scores were significantly attenuated in the block group relative to the control group (all *p* < 0.001). The median intraoperative VAS score was 0.0 [0.0–0.0] in the block group, compared with 6.0 [5.0–7.0] in the control group. This striking analgesic advantage was not transient; rather, it persisted uniformly across all subsequent postoperative assessment intervals.

When applying the clinical pain threshold (VAS ≥ 4), intraoperative clinically significant pain was documented in 25 control patients (83.3%), whereas no patient in the block group reached this threshold (0/30, 0.0%; *p* < 0.001). A parallel narrative emerged during postoperative surveillance. Any clinically significant postoperative pain occurred in 28 patients (93.3%) in the control group and in 1 patient (3.3%) in the block group (*p* < 0.001). Consequently, control allocation carried a substantially higher risk of developing clinically meaningful postoperative pain, with a calculated relative risk of 28.00 (95% CI: 4.07–192.79).

Postoperative sleep quality, assessed using the SQ-NRS on the first postoperative morning, showed lower scores in the block group than in the control group. Specifically, the median SQ-NRS score was 2.0 [1.0–3.0] in the block group versus 7.0 [6.0–8.0] in the control group (*p* < 0.001). Categorical analysis revealed that poor sleep quality (SQ-NRS ≥ 6) afflicted 24 patients (40.0%) overall. Strikingly, this sleep disturbance was entirely isolated to the control group, affecting 80.0% (24/30) of its participants while sparing the block group completely (0/30, 0.0%; *p* < 0.001). Consistent with these results, procedural satisfaction scores were higher for the block group from both clinical and patient perspectives. The median Likert scores for both operating physicians and patients stood at a maximum of 5.0 [5.0–5.0] in the block group, contrasted with a median of 3.0 [3.0–4.0] in the control group (all *p* < 0.001).

The requirement for intraoperative supplemental local anesthesia was lower in the SPSIPB group. The median additional LA requirement was 0.0 [0.0–0.0] mL in the block group versus 1.0 [1.0–2.0] mL in the control group (*p* < 0.001). Viewed as a dichotomous outcome, 23 control patients (76.7%) required supplemental infiltration, whereas the block group required no additional rescue LA (0/30, 0.0%; *p* < 0.001). Mirroring this trend, postoperative systemic analgesic consumption demonstrated a total divergence between the groups (*p* < 0.001). Every patient in the block group remained entirely opioid- and non-opioid-free throughout the 24 h period, whereas 100% of the control patients required at least one rescue analgesic dose. Taken together, these findings suggest a clinically relevant reduction in perioperative pain burden and rescue medication requirements with SPSIPB.

### 3.3. Temporal Evolution of VAS Pain Scores

**Figure 3:**
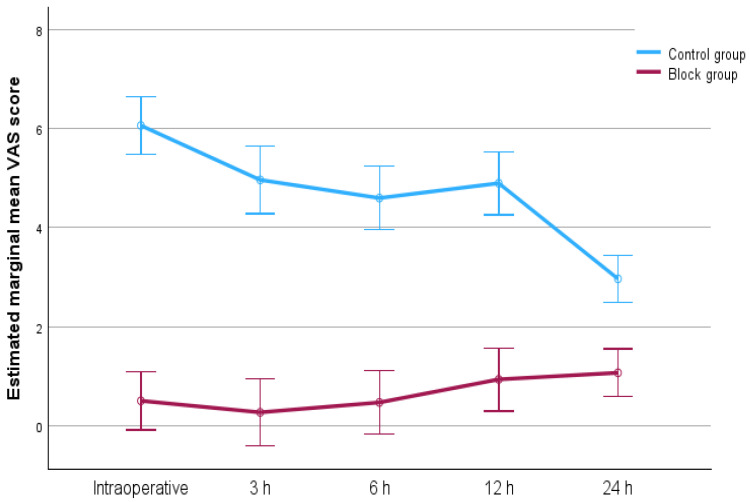
Estimated marginal mean VAS pain scores over time in the control and block groups. Error bars indicate 95% confidence intervals. VAS: visual analog scale.

To rigorously map the longitudinal pain profiles, VAS scores were modeled through a two-way mixed-design repeated-measures analysis of variance (RM-ANOVA), nesting the treatment allocation as the between-subject factor and the longitudinal timeline as the within-subject factor. The temporal evolution of the estimated marginal mean VAS scores across both study groups is graphically illustrated in Figure 3. Structural evaluation via Mauchly’s test revealed a clear violation of the sphericity assumption (W = 0.611, *p* = 0.001). Consequently, the Greenhouse–Geisser correction was implemented to safeguard statistical robustness against inflation of Type I error rates.

The corrected RM-ANOVA models demonstrated a highly significant main effect of time on pain perception, indicating that systemic pain intensity fluctuated dynamically across the five sequential assessment anchors [F(3.266, 189.421) = 5.412, *p* < 0.001]. A significant group-by-time interaction was observed [F(3.266, 189.421) = 11.246, *p* < 0.001], demonstrating that changes in VAS scores over time differed between the SPSIPB and control groups. This interactive divergence is visually underscored by the contrasting geometric trends in Figure 3. Furthermore, a significant main effect of group was observed [F(1, 58) = 299.064, *p* < 0.001], demonstrating consistently lower VAS scores in the SPSIPB group across all assessment time points. Figure 3 illustrates the temporal evolution of the estimated marginal mean VAS scores in both study groups together with their corresponding 95% confidence intervals.

When collapsed across all longitudinal intervals, the overall estimated marginal mean VAS score stood at 4.70 ± 0.17 in the control group versus an ultra-low 0.65 ± 0.17 in the block group, resulting in a substantial overall pooled between-group delta of 4.05 points (95% CI: 3.58 to 4.52; *p* < 0.001). Pairwise comparisons showed that VAS scores were lower in the block group at each assessment point, including the intraoperative period and the 3rd, 6th, 12th, and 24th postoperative hours (all *p* < 0.001). The estimated between-group differences at these time points were 5.57, 4.70, 4.13, 3.97, and 1.90 points, respectively. Overall, these longitudinal findings support a consistent reduction in perioperative pain scores in patients receiving SPSIPB.

### 3.4. Exploratory Analysis of Factors Associated with Pain Outcomes

To uncover potential confounding or contributing elements driving pain experiences, exploratory association models were structured using predetermined VAS summary dimensions: intraoperative VAS, postoperative mean VAS, peak postoperative VAS, the clinical prevalence of postoperative pain (VAS ≥ 4), and time-weighted postoperative area under the curve (VAS-AUC).

Table 4 encapsulates the bivariate correlation matrices linking these summary pain metrics with selected demographic and procedural covariates. Age demonstrated a mild inverse correlation with intraoperative pain scores (ρ = −0.291; *p* = 0.024); however, it failed to maintain any significant cross-correlation with longitudinal postoperative mean VAS, peak postoperative VAS, or calculated VAS-AUC trends. Similarly, BMI, fluoroscopy exposure time, and the technical difficulty of venous puncture displayed no statistically meaningful linkages with postoperative pain configurations. Subcutaneous fat thickness exhibited a weak, negative correlation with time-weighted postoperative VAS-AUC (=−0.268; *p* = 0.039), though its downstream associations with postoperative mean and maximum pain parameters fell short of traditional statistical significance thresholds. Furthermore, splitting the study population by venous puncture difficulty revealed no proportional differences in the absolute frequency of clinically significant postoperative pain [21/44 (47.7%) vs. 8/16 (50.0%); Fisher’s exact *p* = 1.000].

Group-adjusted multi-variable linear regression frameworks for predicting cumulative postoperative VAS metrics are organized in Table 5. Within the baseline model—incorporating treatment assignment, subcutaneous fat thickness, venous puncture difficulty, and BMI—the SPSIPB allocation stood out as the sole independent predictor of attenuated postoperative pain outcomes. For postoperative mean VAS, belonging to the block group predicted an independent 3.659-point reduction in pain scores relative to control counterparts (=−3.659, 95% CI: −4.223 to −3.095; *p* < 0.001). Conversely, subcutaneous fat thickness, venous access difficulty, and BMI exerted no independent predictive influence on either mean or maximum postoperative pain levels. A matching narrative was mirrored for the maximum postoperative VAS model, where block assignment independently commanded a massive 5.344-point drop in peak pain intensity (=−5.344, 95% CI: −6.181 to −4.508; *p* < 0.001).

## 4. Discussion

This randomized sham-controlled trial suggests that SPSIPB may provide effective perioperative analgesia in patients undergoing CIED implantation. Compared with the sham-control group receiving standard local anesthesia, patients in the SPSIPB group had lower intraoperative and postoperative VAS scores during the first 24 h. The intervention was also associated with reduced supplemental local anesthetic use and lower postoperative rescue analgesic requirements. In addition, more favorable SQ-NRS and patient–physician satisfaction scores were observed in the SPSIPB group; however, these secondary outcomes should be interpreted cautiously because the study was not specifically powered for these endpoints. Taken together, these findings support SPSIPB as a promising regional analgesic technique for CIED implantation, while also indicating the need for larger multicenter trials to confirm the magnitude and generalizability of its effects.

Patients undergoing CIED implantation experience pain not only intraoperatively but also throughout the postoperative phase. The perception and expression of pain are highly subjective and specific to each individual. Failure to adequately manage this pain can significantly exacerbate the psychological and emotional burden on patients. Furthermore, insufficient pain control can lead to adverse clinical outcomes, such as an increased risk of immobilization, deliberate avoidance of limb movement on the affected side, prolonged hospital stays, recurrent readmissions, and negative modulatory effects on the immunologic system [28,29,30]. During the implantation procedure itself, involuntary patient movements triggered by acute pain can prolong the operative time and pave the way for various surgical complications. In the postoperative period, the perceived pain can induce undesirable hemodynamic fluctuations by escalating myocardial stress and sympathetic activation, particularly within this fragile patient population characterized by advanced age and multiple cardiac risk factors. Consequently, optimizing perioperative pain management in CIED implantation patients is not merely an ethical obligation but is also critical for accelerating recovery and achieving overall clinical success [7,8].

In recent years, various fascial plane blocks targeting the hemithorax where the generator pocket is to be created have been investigated to achieve effective perioperative analgesia during CIED implantation. Patel et al. administered intra-pocket Pectoral nerve (PECs) I or PECs II blocks to 20 patients undergoing permanent PM or ICD implantation, reporting that 4 patients required opioid rescue within the first 24 postoperative hours [31]. In another study by Zafar et al. evaluating preoperatively performed PECs II blocks in 120 patients, 65% of the participants required supplemental LA and 20% required additional intraoperative opioids [32]. Examining block combinations, Arasu et al. compared a group receiving PECs I alone against a group receiving a combination of PECs I and Transversus Thoracis Muscle (TTM) blocks, demonstrating that the combined approach provided significantly superior analgesia [27]. Furthermore, in a limited-scale study lacking a control group, Antiperovitch et al. noted that combining a supraclavicular nerve block with a PECs I block offered safe and effective analgesia in this patient population [33].

When contrasted with these literature benchmarks, the low requirement for intraoperative supplemental local anesthesia and postoperative analgesics in the SPSIPB group is clinically noteworthy. We hypothesized that a contributing factor behind this clinical finding may be the anatomical spread characteristics of the block. It has been reported that the SPSIPB, originally described by Tulgar et al., achieves LA dissemination across the C7–T7 levels in cadaveric models, and has been reported to produce hemithoracic sensory blockade spanning the C3–T10 dermatomes in clinical cases [14]. Consequently, the SPSIPB may provide a broader dermatomal area compared to traditional PECs blocks, which target more localized anatomical regions. Our findings suggest that this multi-dermatomal spread may help reduce device implantation-induced pain by limiting nociceptive inputs from the skin, subcutaneous tissues, muscles, and the pectoral region via the dorsal rami and the cutaneous branches of the intercostal nerves. Additionally, the execution of the SPSIPB through a single-injection technique may offer a potentially more comfortable alternative for both the patient and the operating physician compared to combination blocks that require multiple needle passes. Beyond its anatomical spread, the analgesic benefit of SPSIPB may also be related to the degree to which it limits nociceptive input from the surgical field. By blocking voltage-gated sodium channels in peripheral sensory nerves, local anesthetics reduce the transmission of impulses arising from skin incision, tissue dissection, and generator-pocket manipulation. More consistent coverage of the relevant intercostal and cutaneous branches may therefore decrease the nociceptive signals carried by Aδ and C fibers. This reduction in afferent input may limit the release of glutamate, substance P, and calcitonin gene-related peptide in the spinal dorsal horn, thereby reducing NMDA receptor-mediated wind-up and the development of central sensitization. It may also attenuate neurogenic inflammation and the release of local inflammatory mediators that contribute to postoperative nociceptor sensitization. In contrast, partial or variable coverage of relevant sensory branches with other fascial plane techniques may allow residual nociceptive input to persist. Although these mechanisms were not directly assessed in the present study, they may provide a biologically plausible explanation for the pronounced analgesic effect observed with SPSIPB [34,35,36].

The magnitude of the observed benefit may also be partly attributable to the highly standardized study setting. SPSIPB was performed preoperatively under ultrasound guidance using a uniform technique, while all procedures were conducted in a single electrophysiology laboratory according to a standardized local anesthetic infiltration protocol. Furthermore, the block was administered by the same experienced anesthesiologist, and all CIED implantations were performed by one experienced electrophysiologist. This consistency may have minimized operator- and protocol-related variability, thereby allowing the analgesic effect of SPSIPB to be more clearly identified. However, the same factors may limit the external validity of our findings. Comparable results may not necessarily be achieved in centers with different levels of expertise in ultrasound-guided regional anesthesia, implantation techniques, perioperative analgesic strategies, or procedural workflows. Variations in block timing, local anesthetic volume and concentration, procedural characteristics, co-analgesic protocols, and outcome definitions may also partly explain why the benefit observed in the present study appeared greater than that reported in previous studies evaluating PECS, ESPB, RIB, or other regional techniques [25,26,27,31,32,33]. Therefore, the reproducibility and clinical magnitude of the analgesic benefit associated with SPSIPB should be evaluated in larger multicentre comparative studies involving different operators and institutional protocols.

The fact that the risk of developing clinically significant postoperative pain was markedly higher in the control group compared to the block group (RR = 28.0, 95% CI: 4.07–192.79) underscores that the SPSIPB is not merely a regional modality that attenuates pain scores, but an intervention that may reduce the occurrence of clinically significant acute postoperative pain. This finding tightly aligns with existing literature evaluating the analgesic efficacy of the SPSIPB in thoracic surgical procedures. For instance, Avci et al. reported that the SPSIPB provided excellent thoracic analgesia in patients undergoing video-assisted thoracoscopic surgery (VATS) [15]. Similarly, a recent study demonstrated that the SPSIPB offers comparable analgesic efficacy to the thoracic paravertebral block, positioning it as a robust alternative within multimodal analgesia protocols [37].

The preoperative administration of SPSIPB may have further enhanced this effect through a preemptive analgesic mechanism. By establishing regional analgesia before surgical trauma, the block may reduce nociceptive afferent input during incision, tissue dissection, and generator-pocket manipulation, thereby limiting the development of central sensitization. This temporal aspect of analgesia may have contributed to the low pain trajectories and reduced analgesic requirements observed in the SPSIPB group. However, as these mechanisms were not directly evaluated, this interpretation should be considered biologically plausible rather than definitive [34,38,39].

In a previous study of ours, we evaluated the clinical efficacy of the preoperative rhomboid intercostal plane block (RIB) in patients undergoing CIED implantation [25]. The outcomes of our current investigation demonstrate that the reduced requirement for intraoperative local anesthesia and postoperative analgesics achieved with SPSIPB appears broadly consistent with the results reported in our previous RIB trial. Crucially, the addition of a formalized assessment regarding the patients’ sleep quality during the first postoperative night in the present study offers a novel clinical perspective. Given its broad multi-dermatomal spread extending across the C3–T10 levels, the SPSIPB may represent a potentially useful option to the RIB in routine clinical practice for CIED implantations.

The profound improvement observed in postoperative sleep quality stands out as one of the most remarkable findings of our investigation. Although sleep quality is a vital patient-centered outcome measure that directly impacts both the physical and psychological dimensions of postsurgical recovery, it is frequently overlooked in regional anesthesia literature [40]. The significantly lower SQ-NRS scores identified in the SPSIPB group in our study demonstrate that the block intervention not only delivers highly effective analgesia but also contributes multi-dimensionally to the overall well-being of the patients. Given that patients for CIED implantation are predominantly elderly individuals with multiple comorbidities, pain-induced sleep fragmentation and its accompanying sympathetic activation can severely jeopardize clinical recovery. At this juncture, evaluating the data against the established threshold for poor sleep quality (SQ-NRS ≥ 6) revealed a striking outcome: while 80% of the patients in the control group suffered from poor sleep quality, not a single patient in the SPSIPB group reached this threshold. This specific metric provides robust proof that the SPSIPB does not merely generate a nominal statistical variance, but possesses a powerful clinical capacity to prevent meaningful sleep disturbances during the critical first postoperative night. Considering the intricate relationship between postoperative pain and sleep quality, the primary mechanism driving this success can be attributed to the long-lasting analgesia provided by the block, which successfully eliminated pain-related nocturnal awakenings. Consequently, the clinical impact of the SPSIPB should be recognized not solely through the lens of pain control, but also through its capacity to preserve sleep quality, which remains an essential component of the postoperative recovery cascade.

Although studies evaluating the relationship between SPSIPB and postoperative quality of recovery remain limited in the literature, existing data corroborate our findings. In a randomized controlled trial conducted by Köksal et al. on patients undergoing breast surgery, it was reported that SPSIPB application led to a reduction in opioid consumption and a significant increase in the quality of recovery, as assessed by the QoR-15 (Quality of Recovery-15) scale [16]. Even though sleep quality was not directly evaluated in their study, the concurrent demonstration of effective analgesia and enhanced recovery quality supports the lower SQ-NRS scores observed in our own trial. Therefore, the benefits of SPSIPB should be evaluated not only through the lens of analgesic efficacy but also in terms of patient-centered recovery outcomes. The lack of any study in the literature examining the impact of SPSIPB on sleep quality following CIED implantation may lend a unique value to our findings.

Higher patient and physician satisfaction scores were also observed in the SPSIPB group. High patient satisfaction is closely linked to the comfortable experience provided during the procedure and throughout the early postoperative period. On the other hand, the marked increase in physician satisfaction may be related to the minimization of involuntary patient movements caused by pain during the procedure, the elimination of additional analgesic/sedative requirements, and the consequent provision of a more controlled, predictable working environment [27]. Although there are no studies in the literature evaluating the effect of SPSIPB on physician satisfaction, the results of our study demonstrate that this block can offer potential benefits not just for analgesic efficacy, but also for overall procedural quality and the perioperative experience.

When evaluating the findings of our investigation, certain limitations must be taken into consideration. First, because this research was conducted at a single tertiary care center with a relatively small sample size, generalizing our conclusions to diverse institutional settings and wider patient populations may be constrained. In addition, SPSIPB was performed by one experienced anesthesiologist, and all CIED implantation procedures were conducted by one experienced electrophysiologist using a highly standardized procedural and local anesthetic infiltration protocol. Although this approach reduced operator- and protocol-related variability, it may limit the reproducibility and generalizability of the observed effect size in centers with different levels of ultrasound-guided regional anesthesia expertise, implantation techniques, perioperative analgesic practices, or procedural workflows. Second, although the study was adequately powered to detect differences in our primary outcome, it lacked a specific, dedicated statistical power calculated to definitively detect variances among secondary outcomes, such as sleep quality and participant or clinician satisfaction scores. Third, the sample size calculation was based on a pragmatic large-effect assumption derived from indirect evidence, because no previous SPSIPB trial in CIED implantation was available. Accordingly, the observed magnitude of effect should be interpreted with caution, particularly given the single-center design and the standardized procedural setting. In addition, secondary outcomes such as postoperative sleep quality and patient–physician satisfaction were not supported by separate power calculations. No formal adjustment for multiplicity was applied for secondary and exploratory comparisons; therefore, these findings should be regarded as exploratory and hypothesis-generating. Finally, the 24 h follow-up period precluded evaluation of persistent pocket pain, longer-term functional recovery, chronic post-implantation pain, and delayed procedure-related complications. Accordingly, whether the analgesic benefits of SPSIPB are sustained beyond the immediate postoperative period warrants confirmation in future studies with longer follow-up.

## 5. Conclusions

In this single-center randomized sham-controlled trial, preoperative SPSIPB was associated with lower perioperative VAS scores and reduced rescue analgesic requirements during the first 24 h after CIED implantation. Favorable differences in postoperative SQ-NRS and patient–physician satisfaction scores were also observed, although these secondary outcomes should be interpreted as exploratory. SPSIPB may represent a promising regional analgesic technique for CIED implantation; however, larger multicenter studies are needed to confirm the magnitude, reproducibility, and clinical generalizability of these findings.

## Figures and Tables

**Table 1:** Baseline demographic and clinical characteristics.

	Overall (*n* = 60)	Control Group (*n* = 30)	Block Group (*n* = 30)	*p* Value
Age, years	64.5 [58.3–75.8]	61.5 [51.3–74.5]	70.5 [60.8–77.3]	0.071
Sex, *n* (%):				0.121
Female	28 (46.7%)	11 (36.7%)	17 (56.7%)	
Male	32 (53.3%)	19 (63.3%)	13 (43.3%)	
BMI, kg/m^2^	26.3 ± 4.2	25.9 ± 4.2	26.7 ± 4.2	0.486
Education status, *n* (%):				0.200
Primary school	34 (56.7%)	16 (53.3%)	18 (60.0%)	
Secondary school	11 (18.3%)	6 (20.0%)	5 (16.7%)	
High school	9 (15.0%)	7 (23.3%)	2 (6.7%)	
University	5 (8.3%)	1 (3.3%)	4 (13.3%)	
No formal education/none	1 (1.7%)	0 (0.0%)	1 (3.3%)	
Indication, *n* (%)				0.404
Arrhythmia	35 (58.3%)	17 (56.7%)	18 (60.0%)	
Ischemic cardiomyopathy	12 (20.0%)	8 (26.7%)	4 (13.3%)	
Non-ischemic cardiomyopathy	10 (16.7%)	5 (16.7%)	5 (16.7%)	
Arrhythmia + non-ischemic cardiomyopathy	1 (1.7%)	0 (0.0%)	1 (3.3%)	
Genetic + non-ischemic cardiomyopathy	2 (3.3%)	0 (0.0%)	2 (6.7%)	

Values are presented as mean ± SD, median [Q1–Q3], or *n* (%), as appropriate. Percent ages are column percentages. BMI, body mass index.

**Table 2:** Procedural characteristics.

Variable	Overall (*n* = 60)	Control Group (*n* = 30)	Block Group (*n* = 30)	*p* Value
Access vein, *n* (%):				0.606
Axillary	30 (50.0%)	14 (46.7%)	16 (53.3%)	
Subclavian	30 (50.0%)	16 (53.3%)	14 (46.7%)	
Device type, *n* (%):				0.230
CRT-D	14 (23.3%)	5 (16.7%)	9 (30.0%)	
Dual-chamber ICD (DR-ICD)	26 (43.3%)	17 (56.7%)	9 (30.0%)	
Dual-chamber pacemaker (DR-PM)	16 (26.7%)	7 (23.3%)	9 (30.0%)	
Single-chamber ICD (VVIR-ICD)	3 (5.0%)	1 (3.3%)	2 (6.7%)	
Single-chamber pacemaker (VVIR-PM)	1 (1.7%)	0 (0.0%)	1 (3.3%)	
Number of leads	2.0 [2.0–2.0]	2.0 [2.0–2.0]	2.0 [2.0–3.0]	0.495
Venous puncture difficulty, *n* (%):				0.559
Easy	44 (73.3%)	21 (70.0%)	23 (76.7%)	
Difficult	16 (26.7%)	9 (30.0%)	7 (23.3%)	
CIED implantation side, *n* (%):				1.000
Left	58 (96.7%)	29 (96.7%)	29 (96.7%)	
Right	2 (3.3%)	1 (3.3%)	1 (3.3%)	
Battery placement, *n* (%):				0.795
Subfascial	27 (45.0%)	13 (43.3%)	14 (46.7%)	
Subpectoral	33 (55.0%)	17 (56.7%)	16 (53.3%)	
Fluoroscopy time, min	12.5 [9.0–19.0]	12.5 [8.4–19.6]	12.9 [9.1–19.7]	0.882
Subcutaneous fat thickness, cm	0.7 ± 0.1	0.7 ± 0.2	0.7 ± 0.1	0.466
Intraoperative total LA dose, mL	20.0 [20.0–25.0]	25.0 [23.8–30.0]	20.0 [20.0–20.0]	<0.001
Any complication, *n* (%):				1.000
No	53 (88.3%)	26 (86.7%)	27 (90.0%)	
Yes	7 (11.7%)	4 (13.3%)	3 (10.0%)	

Values are presented as mean ± SD, median [Q1–Q3], or *n* (%), as appropriate. Percentages are column percentages. CIED, cardiac implantable electronic device; CRT-D, cardiac resynchronization therapy-defibrillator; DR-ICD, dual-chamber implantable cardioverter-defibrillator; DR-PM, dual-chamber pacemaker; LA, local anesthetic.

**Table 3:** Pain, sleep quality, satisfaction, and analgesic outcomes.

Variable	Overall (*n* = 60)	Control Group (*n* = 30)	Block Group (*n* = 30)	*p* Value
VAS intraoperative	3.0 [0.0–6.0]	6.0 [5.0–7.0]	0.0 [0.0–0.0] *	<0.001
VAS 3rd hour	2.0 [0.0–4.0]	4.0 [3.0–7.0]	0.0 [0.0–0.0] *	<0.001
VAS 6th hour	2.0 [0.0–3.5]	4.0 [3.0–7.0]	0.0 [0.0–0.0] *	<0.001
VAS 12th hour	2.0 [0.0–5.0]	5.0 [3.0–6.8]	0.0 [0.0–2.0] *	<0.001
VAS 24th hour	2.0 [0.0–3.0]	3.0 [2.0–3.0]	0.0 [0.0–2.0] *	<0.001
Clinically significant intraoperative pain, VAS ≥ 4, *n* (%)	25 (41.7%)	25 (83.3%)	0 (0.0%)	<0.001
Any clinically significant postoperative pain, VAS ≥ 4, *n* (%)	29 (48.3%)	28 (93.3%)	1 (3.3%)	<0.001
SQ-NRS (09:00)	3.0 [2.0–7.0]	7.0 [6.0–8.0]	2.0 [1.0–3.0]	<0.001
Poor postoperative sleep quality, SQ-NRS ≥ 6, *n* (%)	24 (40.0%)	24 (80.0%)	0 (0.0%)	<0.001
Likert score, physician	4.0 [3.0–5.0]	3.0 [3.0–4.0]	5.0 [5.0–5.0]	<0.001
Likert score, patient	4.0 [3.0–5.0]	3.0 [3.0–4.0]	5.0 [5.0–5.0]	<0.001
Additional local anesthetic requirement	0.0 [0.0–1.0]	1.0 [1.0–2.0]	0.0 [0.0–0.0]	<0.001
Any additional local anesthetic requirement, *n* (%)	23 (38.3%)	23 (76.7%)	0 (0.0%)	<0.001
Any postoperative analgesia, *n* (%)	30 (50.0%)	30 (100.0%)	0 (0.0%)	<0.001
Paracetamol requirement, *n* (%)	30 (50.0%)	30 (100.0%)	0 (0.0%)	<0.001
Tramadol requirement, *n* (%)	14 (23.3%)	14 (46.7%)	0 (0.0%)	<0.001

Values are presented as median [Q1–Q3] or *n* (%), as appropriate. Percentages are column percentages. LA, local anesthetic; SQ-NRS, sleep quality numeric rating scale; VAS, visual analog scale. Clinically significant pain was defined as VAS ≥ 4. Any clinically significant postoperative pain was defined as VAS ≥ 4 at least once at the 3rd, 6th, 12th, or 24th postoperative hour. *p* values were calculated using Pearson’s chi-square test or Fisher’s exact test, as appropriate. * Although a few patients in the block group reported non-zero VAS scores, the median and interquartile range remained 0.0 [0.0–0.0] at the intraoperative, 3rd-hour, and 6th-hour assessments because most patients had no pain. At the 12th and 24th hours, the upper quartile increased to 2.0, suggesting mild residual pain in a subset of patients.

**Table 4:** Spearman correlation analysis between VAS summary measures and selected demographic and procedural variables.

Variable	Intraoperative VAS Rho (ρ)	Postoperative Mean VAS Rho (ρ)	Maximum Postoperative VAS Rho (ρ)	Postoperative VAS-AUC Rho (ρ)
Age	−0.291 (0.024)	−0.127 (0.333)	−0.132 (0.314)	−0.130 (0.324)
BMI	0.000 (0.999)	−0.054 (0.683)	−0.040 (0.761)	−0.111 (0.398)
Subcutaneous fat thickness	−0.017 (0.898)	−0.200 (0.126)	−0.167 (0.201)	−0.268 (0.039)
Fluoroscopy time	0.090 (0.494)	0.019 (0.885)	0.018 (0.891)	0.006 (0.963)
Venous puncture difficulty	0.194 (0.137)	0.005 (0.967)	0.028 (0.834)	−0.003 (0.980)

Values are presented as Spearman’s rho coefficients with corresponding *p* values. VAS, visual analog scale; AUC, area under the curve; BMI, body mass index.

**Table 5:** Group-adjusted associations of subcutaneous fat thickness, venous puncture difficulty, and BMI with postoperative VAS outcomes.

Outcome	Predictor	B	95% CI	*p* Value
Postoperative mean VAS	Block group	−3.659	−4.223 to −3.095	<0.001
	Subcutaneous fat thickness	−0.758	−2.879 to 1.364	0.477
	Difficult venous puncture	−0.066	−0.724 to 0.593	0.842
	BMI	0.002	−0.074 to 0.077	0.967
Maximum postoperative VAS	Block group	−5.344	−6.181 to −4.508	<0.001
	Subcutaneous fat thickness	−0.474	−3.620 to 2.673	0.764
	Difficult venous puncture	−0.243	−1.220 to 0.733	0.619
	BMI	0.010	−0.102 to 0.123	0.854

Linear regression models were adjusted for treatment group, subcutaneous fat thickness, venous puncture difficulty, and BMI. Regression coefficients for subcutaneous fat thickness refer to a 1 cm increase. VAS, visual analog scale; BMI, body mass index; CI, confidence interval.

## Data Availability

The data presented in this study are available on reasonable request from the corresponding author.

## Supplementary Materials

The following supporting information can be downloaded at: https://www.mdpi.com/article/10.3390/jcm15145629/s1, CONSORT 2025 checklist of information to include when reporting a randomised trial.

## Abbreviations

The following abbreviations are used in this manuscript:

ASA	American Society of Anesthesiologists
BMI	Body Mass Index
CCU	Coronary Care Unit
CIED	Cardiac Implantable Electronic Device
CONSORT	Consolidated Standards of Reporting Trials
CRT	Cardiac Resynchronization Therapy
CRT-D	Cardiac Resynchronization Therapy Defibrillator
DR-ICD	Dual-Chamber Implantable Cardioverter-Defibrillator
DR-PM	Dual-Chamber Pacemaker
ICD	Implantable Cardioverter-Defibrillator
LA	Local Anesthetic
NMDA	N-metil-D-aspartat
NYHA	New York Heart Association
PM	Pacemaker
RM-ANOVA	Repeated-Measures Analysis of Variance
SPS	Serratus Posterior Superior
SPSIPB	Serratus Posterior Superior Intercostal Plane Block
SQ-NRS	Sleep Quality Numeric Rating Scale
VAS	Visual Analog Scale
VAS-AUC	Area Under the Curve of Visual Analog Scale Scores
VVIR-ICD	Single-Chamber Implantable Cardioverter-Defibrillator
VVIR-PM	Single-Chamber Pacemaker
